# Impact of mobile technologies on cervical cancer screening practices in Lagos, Nigeria (mHealth-Cervix): Protocol for a randomised controlled trial

**DOI:** 10.12688/f1000research.22991.1

**Published:** 2020-05-04

**Authors:** Kehinde S. Okunade, Omolola Salako, Adebola A. Adejimi, Oluwatosin J. Akinsola, Omolara Fatiregun, Muisi A. Adenekan, Olusanjo E. Moses, Bassey Ebenso, Matthew J. Allsop, Rose I. Anorlu, Jonathan S. Berek

**Affiliations:** 1 Department of Obstetrics and Gynaecology, University of Lagos, Lagos, Lagos, 2345, Nigeria; 2 Department of Radiation Biology, Radiotherapy and Radiodiagnosis, University of Lagos, Lagos, 2345, Nigeria; 3 Department of Community Health and Primary Care, University of Lagos, Lagos, Lagos, 2345, Nigeria; 4 Department of Clinical and Radiation Oncology, Lagos State University Teaching Hospital,, Ikeja, Lagos, 2345, Nigeria; 5 Department of Obstetrics and Gynaecology, Lagos University Teaching Hospital,, Lagos, Lagos, 2345, Nigeria; 6 Department of Obstetrics and Gynaecology, State University Teaching Hospital, Ikeja, Lagos, 2345, Nigeria; 7 Nuffield Centre for International Health and Development, University of Leeds, Leeds, LS2 9JT, UK; 8 Academic Unit of Palliative Care, University of Leeds, Leeds, LS2 9JT, UK; 9 Stanford Women’s Cancer Center, Stanford Cancer Institute, Stanford University School of Medicine, Palo Alto, California, 94304, USA

**Keywords:** Cervical cancer, Lagos, mHealth-Cervix, NHIS, Nigeria, Pap test

## Abstract

**Background:** Incidence and mortality from cervical cancer have remained high due to many obstacles facing the implementation of organized screening programs in resource-constrained countries such as Nigeria. The application of mobile technologies (mHealth) to health services delivery has the potential to reduce inequalities, empower patients to control their health, and improve the cost-effectiveness of health care delivery.

**Aim:** To assess the efficacy of mobile technology intervention on Pap test screening adherence compared to a control condition and also determine the factors affecting the uptake of Pap smear screening practices among women in Lagos.

**Methods:** This is a multi-center randomized controlled trial that will involve women aged 25 to 65 years attending the General Outpatient clinics of the two tertiary health institutions in Lagos, Nigeria between April and December 2020. At baseline, a total of 200 National Health Insurance Scheme (NHIS) enrollees will be randomized to either a text message arm or usual care (control) arm. The primary outcome is the completion of a Pap smear within 6 months of enrolment in the study. The associations between any two groups of continuous variables will be tested using the independent sample t-test (normal distribution) or the Mann-Whitney U test (skewed data) and that of two groups of categorical variables with Chi-square *X ^2^*or Fisher's exact test where appropriate. Using binary logistic regression model, we will adjust for age and other relevant sociodemographic and clinical variables and adherence to Pap test screening. Statistical significance will be defined as *P*-value less than 0.05.

**Discussion: **The mHealth-Cervix study will evaluate the impact of mobile technologies on cervical cancer screening practices in Lagos, Nigeria as a way of contributing to the reduction in the wide disparities in cervical cancer incidence through early detection facilitated using health promotion to improve Pap smear screening adherence.

**Registration**: PACTR202002753354517 13/02/2020

## Introduction

Cervical cancer is a major public health problem and is the fourth most common cancer in women worldwide accounting for an estimated 570,000 new cases annually ^1^. More than 80% of the global burden of cervical cancer occurs in the less developed regions, where it accounts for almost 12% of all female malignancies ^2, 3^. In 2018, an estimated 311,000 deaths were attributed to cervical cancer, which accounts for 7.5% of all female cancer deaths with 70% of these occurring in developing countries ^1^. Early detection of precursor lesions of cervical cancer through the use of screening tests has drastically reduced the incidence of the disease, especially in Western countries where Pap smear (cytology-based) screening has been introduced and now covers almost all eligible women ^4^. However, in resource-constrained countries of the world such as Nigeria, cervical cancer incidence and mortality have remained high due to the many obstacles facing the implementation of organized screening programs ^5^. A recent study conducted by our team among women in Lagos revealed Pap smear screening uptake of about 23% despite an awareness rate of 55% ^6^.

The use of mobile technologies has increased exponentially in the last few years ^7^. We reported in a study conducted in 2018 that mobile telephones could be found in 95% of households in Lagos and these were widely distributed across all socioeconomic classes ^8^. Because of this increased access to technologies, mobile health (mHealth), or “medical and public health practice involving the use of mobile devices” ^9^, has great potential in many health areas such as promotion and prevention ^10^. The World Health Organization (WHO) report on mHealth in 2011 ^9^ states that mobile health strategies exist in at least 75% of the countries in WHO regions. The application of mHealth to health services delivery has the potential to reduce inequalities, empower patients to control their health, and improve the cost-effectiveness of health care delivery ^11^.

Although still limited, there is growing evidence of success in the use of mobile phones for health (mHealth technologies) to support cancer prevention ^12– 15^. Nevertheless, there is currently no reported randomized controlled trial in sub-Saharan Africa (sSA) that examines the use of mobile health technologies in cancer prevention. This study will, therefore, aim to ascertain the efficacy of an intervention using mobile technologies on Pap smear screening adherence compared to standard care; and also determine the factors affecting the uptake of Pap smear screening services among women in Lagos, Nigeria. The introduction of mHealth, now regarded as one of the most promising investments for health in developing countries ^16^, is a key innovative concept that will offer a unique opportunity for a paradigm shift in cervical cancer control in a resource-constrained setting like Nigeria and other parts of sSA.

## Protocol

### Study design

This study (mHealth-Cervix) is a multi-center randomized controlled trial that will involve women aged 25 to 65 years who are attending the General Outpatient (GOP) clinics of two public tertiary health institutions in Lagos, Nigeria – Lagos University Teaching Hospital (LUTH), Idi-Araba and Lagos State University Teaching Hospital (LASUTH), Ikeja between April and December 2020. The trial protocol has been registered in the Pan African Clinical Trial Registry (PACTR) ( PACTR202002753354517; version 3.0; February 13, 2020).

### Study settings

LUTH and LASUTH are the two foremost public tertiary health institutions in Lagos that offer speciality and sub-speciality care including gynaecological oncology services such as Papanicolaou (Pap) test, human papillomavirus (HPV) testing and colposcopy for cervical cancer screening. The GOP clinics of the two hospitals run from Monday to Friday each week and attendees at the clinics are mostly enrollees of the National Health Insurance Scheme (NHIS) who have free cervical cancer screening as part of their health coverage. The hospitals act mainly as referral centers for other government-owned and private hospitals in Lagos State. Lagos State is the commercial capital of Nigeria which has a population of over 9 million inhabitants.

### Study population and eligibility criteria

Eligible women are NHIS enrollees aged between 25 and 65 with no prior history of cervical cancer or cervical dysplasia; those not adherent with current recommendations for Pap smear screening (have not had a Pap smear within the last three years); those owning and using a personal cell-phone; those free from any mental or physical disabilities that inhibit them from understanding the implications of the study and those not considering relocating from their current residence within the next year. The exclusion criteria include women younger than 25 and older than 65 years old, those with history of cervical dysplasia and cancer, those who had a Pap test within the last three years, those with an ongoing pregnancy and those who refuse or withdraw consent during the study.

### Participants’ enrollment and data collection

Investigators will identify eligible women from the GOP clinics of the two study sites on each day of the study period after which a 20–30 minutes educational health talk on cervical cancer and its prevention is given to these women by the clinic midwives with the assistance of the clinics’ health care teams as part of their usual standard of care. Eligible women are then invited by the investigators to give consent for participation in the clinical trial upon explanation of the purpose and nature of the study. Once consent is obtained, an interviewer-administered questionnaire (see extended data ^17^) is then used to obtain baseline information on sociodemographic variables, cell-phone use, and distance of participants’ residence from the clinics (measured in kilometer using the Google maps). Each woman will receive an estimated $5 credit charge on their cellphones as a token to enable the investigators to keep their phone numbers for as long as the intervention lasts. The women in both arms are each given the complementary card containing the cell-phone number of the cytology clinic coordinators (who are also part of the study) and are subsequently scheduled for 6-month follow-up appointments from their dates of study enrolment. After follow-up, data will be collected from each woman on completion of Pap test screening, the number of GOP clinics attended, the interval from enrollment to date of Pap smear testing and functionality of cell-phone during the same follow-up period (that is, if the cell-phone was in use for at least 5 out the 6 months follow-up or not).

### Randomization

**Figure 1.**
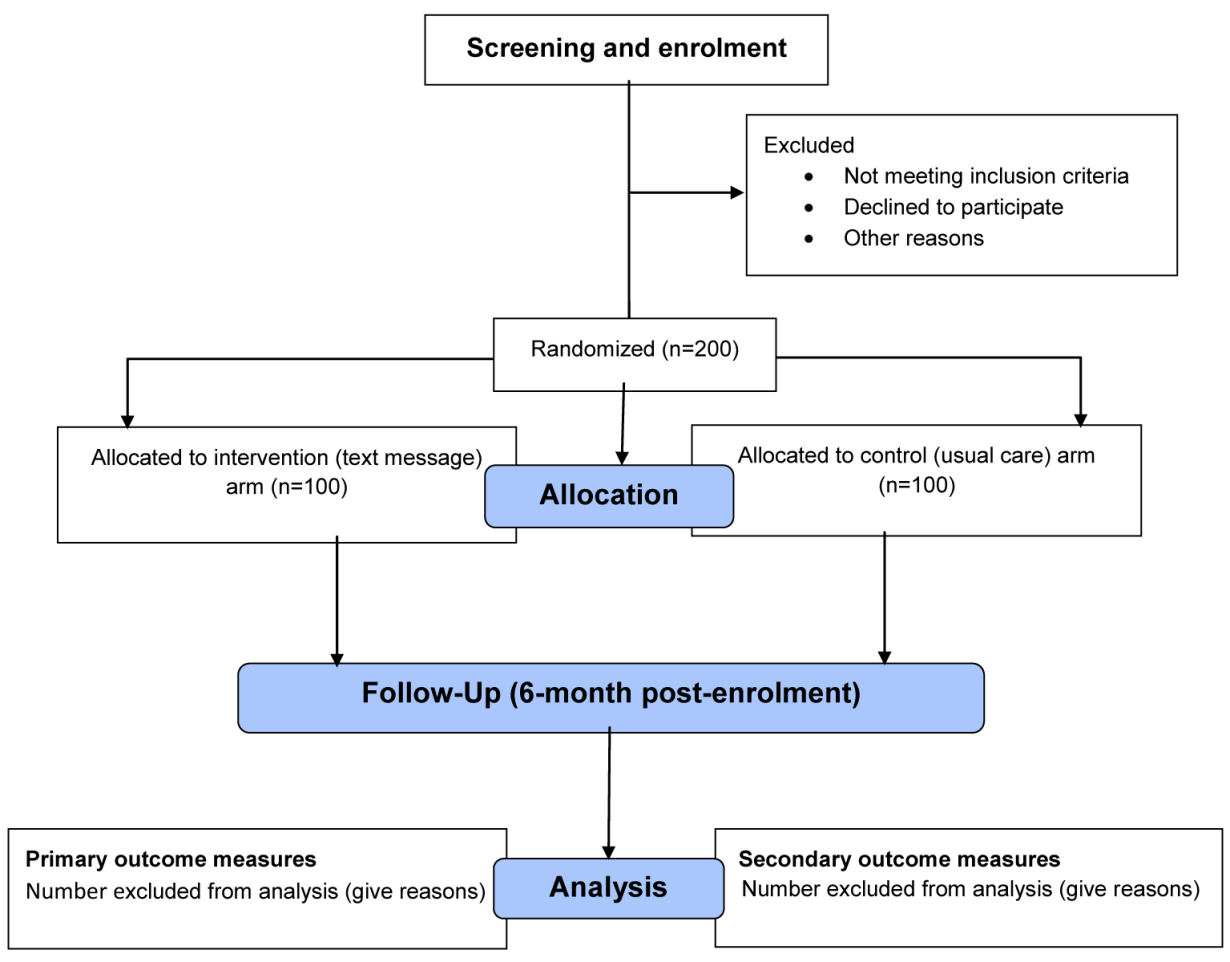
Trial flow chart.

Following the baseline assessment, enrolled women will be randomized to either a text message (intervention) arm or usual care (control) arm using computer-generated block randomization codes using Random Allocation software version 1.0 (May 2004) by a study statistician. Investigators and statisticians will be blinded to the allocation groups [ Figure 1]. The allocation sequence is concealed in sealed opaque envelopes that will be kept in a locked file cabinet at each of the study sites until participants assignment is completed.

*Intervention (mHealth) arm* – We chose Nexmo® as the platform to deliver the mHealth messages given its low cost and reliability. Participants will be sent messages containing information and encouragement to undergo cervical cancer screening. Information will also be provided about the Cytology clinic hours, contact information and locations in the two participating hospitals. Information and motivational text messages will be delivered twice monthly for the next 6-months after enrollment.

*Usual care (control) arm* – Participants randomized to the control arm will not receive additional reminder messages from study staff other than the usual care received at enrollment. Usual care consists of any information on Pap tests and cervical cancer risk reduction typically provided by midwives to all women at the clinics as will be done at enrollment. These women can then schedule an appointment for their Pap smear testing in person at the GOP or Cytology clinics at any time during the 6-month follow-up period.

### Study endpoints

Primary endpoint – This is the completion of a Pap smear within 6 months of enrollment in the study. Participants will be tracked via medical record review as well as through phone calls at 6-month after their enrolment.

Secondary endpoint – This is an assessment of the various factors affecting Pap smear uptake or adherence within 6-month of enrollment among the participating women.

### Statistical methods

Sample size – We calculated sample size using G*Power for Windows version 3.1.9.2 (Kiel University, Germany). Using data from our published study ^6^, we estimated the proportion of Pap smear adherence to be approximately 23% for the usual care (control) arm and 60% for the text message (intervention) arm. This is powered for a two-sided test with a Type I error rate of 5% and 80% power, i.e. Zα=1.96 and Zβ=0.84, adjusted for a 10% attrition rate. Therefore, the power calculations will be based on a sample size of 100 in each of the two study arms (making a total sample size of 200).

Statistical analysis – The intention-to-treat principle will be used in the final analyses. Statistical analyses will be carried out using SPSS version 23.0 for Windows (Armonk, NY: IBM Corp.). Pap smear adherence will be coded as a binary variable. The intervention will be evaluated based only on Pap smear completion at 6-month after the enrollment and baseline survey. The associations between any two groups of continuous variables will be tested using the independent sample t-test (normal distribution) or the Mann-Whitney U test (skewed data) and that of two groups of categorical variables with Chi-square ( *χ* ^2^) or Fisher's exact test where appropriate. Using binary logistic regression analysis, we will adjust for age, socioeconomic class, parity, marital status, number of GOP clinic attendance, the distance of residence from the clinics, interval from enrollment to date of Pap smear testing, the functionality of cellphones and other relevant demographic and clinical variables. Statistical significance will be defined as *P*-value less than 0.05.

### Quality control and data monitoring

All investigators and research assistants will be required to undergo training before the trial to guarantee consistent practice. The training will include an understanding of inclusion/exclusion criteria, follow-up procedures, and completion of the questionnaire. Identifiable data will be transferred to an electronic database system located in a guarded facility at the trial site by the research assistant. Access to identifiable data is restricted only to the PI during and after the trial completion. The trial will be monitored by quality assurance personnel from the research management office of the College of Medicine, University of Lagos, who will be independent of the study team, and an independent steering committee. There will be periodic monitoring to guarantee accuracy and quality throughout the study period. The essential documents (consent information, enrolment, protocol deviations, and losses to follow-up) will be monitored and checked for accuracy and completeness by the monitors. The principal investigator (KSO) is responsible for the overall project and for organizing steering committee meetings. The independent steering committee will be responsible for ensuring the overall safety of participants, coordinating study meetings, supervising the study, monitoring data safety, and overseeing quality control.

### Ethics

This study protocol was approved by the Health Research Ethics Committee of College of Medicine, University of Lagos (CMUL HREC) on 2 ^nd^ February 2020 with approval number CMUL/HREC/12/19/704. Participants will provide written informed consent before their participation in the trial and the participant will keep a signed copy of the consent form for future references.

### Dissemination of information

The trial will be reported in line with the Consolidated Standards of Reporting Trials (CONSORT) checklist and the results will be disseminated in peer-review scientific journals. Important modifications to the trial protocol will be communicated to the funder, study investigators, CMUL HREC, trial participants and trial registries.

### Study status

At the time of initial manuscript submission, recruitment is yet to commence into the trial. Participants’ enrolment will start in May 2020, and the last woman is expected to be included in the trial in July 2020. The manuscript reports protocol version 3.0 (30 ^th^ March 2020).

## Discussion

Cervical cancer is a major public health disease in Nigeria. However, screening is still not a national priority as only 23% of adult females has ever been screened ^6^. There is wide use of mobile technologies in Nigeria and most parts of sub-Saharan Africa, but there are currently no randomized controlled trials that have described the use of these technologies for cancer screening. This protocol describes a randomised controlled trial of a novel intervention (mHealth technologies using text messages) to improve Pap smear screening adherence among women in Lagos. The mHealth-Cervix study will evaluate the impact of mobile technologies on cervical cancer screening practices in Lagos, Nigeria and determine the factors that affect this screening adherence as a means of reducing the disparities in the incidence of cervical cancer through early detection facilitated using health promotion to improve Pap smear screening adherence. If found to be effective in increasing adherence to screening, the mHealth intervention strategy may become an important tool for reducing the cervical cancer burden, and its associated morbidity and mortality. This could also be applied in the future for the health promotion and prevention of other major disease conditions.

## Data availability

### Underlying data

No data are associated with this article

### Extended data

Harvard Dataverse: Replication Data for: Impact of mobile technologies on cervical cancer screening practices in Lagos, Nigeria (mHealth-Cervix): Protocol for a randomised controlled trial https://doi.org/10.7910/DVN/GXE1ON ^17^

*This trial protocol contains the following underlying data:*

- *Consent form.pdf (Study consent form)*
- *Questionnaire_f1000.docx*

### Reporting guidelines

SPIRIT checklist for “Impact of mobile technologies on cervical cancer screening practices in Lagos, Nigeria (mHealth-Cervix): Protocol for a randomised controlled trial” https://doi.org/10.7910/DVN/GXE1ON ^17^

Data are available under the terms of the Creative Commons Zero "No rights reserved" data waiver (CC0 1.0 Public domain dedication).
