# Supplementary material for: Impact of mobile technologies on cervical cancer screening practices in Lagos, Nigeria (mHealth-Cervix): Protocol for a randomised controlled trial
Source: F1000Res. 2020 May 4;9:322. [Version 1] doi: 10.12688/f1000research.22991.1 (PMC7265590; doi:10.12688/f1000research.22991.1)
Supplement: Peer review history

# Peer review: Reviewer response for version 1

**DOI:** 10.5256/f1000research.25380.r63096

**Published:** 2020-06-01

**Authors:** Lateef Akinola

**Affiliations:** 1 Medison Specialist Women's Hospital and Fertility Assyst, Lagos, Nigeria

The article 'Impact of technologies on cervical cancer screening practices in Lagos, Nigeria (mHealth Cervix): Protocol for a RCT by Okunade KS *et al.*, set out to understudy a very relevant and timely aspect on cancer preventive initiative in Nigeria specifically in Lagos the economic hub and most populous city of Nigeria. This is aimed at preventing the incidence and mortality from cervical cancer in the urban city of Lagos in Nigeria. The methodology to study MHealth Cervix in Lagos is excellent and will cover the relevant population of Lagos city. The power 80% is significant enough and the sample size is robust. The statistical analysis package is more than adequate to analyse the research result specifically by using the two tests for comparison of means or test of significance. The research report is anticipated and I hope the author will proceed to the more predictive test using HPV screening in their future research on this very important subject matter.

Is the study design appropriate for the research question?

Yes

Is the rationale for, and objectives of, the study clearly described?

Yes

Are sufficient details of the methods provided to allow replication by others?

Yes

Are the datasets clearly presented in a useable and accessible format?

Yes

Reviewer Expertise:

Obstetrics and gynaecology, reproductive medicine and surgery specialist

I confirm that I have read this submission and believe that I have an appropriate level of expertise to confirm that it is of an acceptable scientific standard.

# Peer review: Reviewer response for version 1

**DOI:** 10.5256/f1000research.25380.r63099

**Published:** 2020-05-13

**Authors:** Adeola Olaitan

**Affiliations:** 1 Gynaecological Cancer Centre, University College London Hospital, London, UK

The research addresses the importance of improving compliance with cervical screening using mobile phone technology. I am not familiar with the platform described and would suggest another reviewer comments on the appropriateness.

 This manuscript presents methods but no results yet. I suggest the paper will make for more interesting reading when results are available. Also the endpoints of the study are attendance for screening. As the purpose of screening is to prevent cervical cancer, a more important goal will be difference in outcome between the two study groups in terms of detection rates of premalignant lesions and eventual outcome. I appreciate the numbers may be too small to demonstrate a reduction in cervical cancer.

 Finally I question why cervical smears are the chosen screening methods when all available data indicate that HPV testing is more reliable and objective and is indeed the method of choice in the national cancer plan.

Is the study design appropriate for the research question?

Yes

Is the rationale for, and objectives of, the study clearly described?

Yes

Are sufficient details of the methods provided to allow replication by others?

Yes

Are the datasets clearly presented in a useable and accessible format?

Partly

Reviewer Expertise:

NA

I confirm that I have read this submission and believe that I have an appropriate level of expertise to confirm that it is of an acceptable scientific standard.
